# Vision/INS Integrated Navigation System for Poor Vision Navigation Environments

**DOI:** 10.3390/s16101672

**Published:** 2016-10-12

**Authors:** Youngsun Kim, Dong-Hwan Hwang

**Affiliations:** 1 Payload Electronics Team, Korea Aerospace Research Institute, Daejon 34133, Korea; yskim1203@kari.re.kr; 2 Department of Electronics Engineering, Chungnam National University, Daejon 34134, Korea

**Keywords:** vision navigation system, inertial navigation system, integrated navigation, focal plane measurements, landmark

## Abstract

In order to improve the performance of an inertial navigation system, many aiding sensors can be used. Among these aiding sensors, a vision sensor is of particular note due to its benefits in terms of weight, cost, and power consumption. This paper proposes an inertial and vision integrated navigation method for poor vision navigation environments. The proposed method uses focal plane measurements of landmarks in order to provide position, velocity and attitude outputs even when the number of landmarks on the focal plane is not enough for navigation. In order to verify the proposed method, computer simulations and van tests are carried out. The results show that the proposed method gives accurate and reliable position, velocity and attitude outputs when the number of landmarks is insufficient.

## 1. Introduction

The inertial navigation system (INS) is a self-contained dead-reckoning navigation system that provides continuous navigation outputs with high-bandwidth and short-term stability. Due to its navigation characteristics, the accuracy of the navigation output degrades as time passes. In order to improve the performance of the INS, a navigation aid can be integrated into the INS. The GPS/INS integrated navigation system is one of the most generally used integrated navigation systems [1,2]. However, the GPS/INS integrated navigation system may not produce reliable navigation outputs, since the GPS signal is vulnerable to interference such as jamming and spoofing [3,4]. In recent years, many alternative navigation systems to GPS such as vision, radar, laser, ultrasonic sensor, UWB (Ultra-Wide Band) and eLoran (enhanced Long range navigation) have been studied in order to provide continuous, reliable navigation outputs [4].

Vision sensors have recently been used for navigation of vehicles such as cars, small-sized low-cost airborne systems and mobile robots due to their benefits in terms of weight, cost and power consumption [5,6,7]. Navigations using vision sensors can be classified into three methods [4,7]. The first method determines the position of the vehicle by comparing the measured image of a camera with the stored image or stored information of a map [8]. The second method, which is called landmark-based vision navigation, determines position and attitude by calculating directions to landmarks from the measured image of the landmarks [9,10]. The third method, called visual odometry, determines the motion of the vehicle from successive images of the camera [11]. Among these three methods, the landmark-based approach is known to have the advantages of bounded navigation parameter error and simple computation [7].

In order to integrate an inertial navigation system with a vision navigation system, several methods have been proposed [12,13,14,15,16]. The method in [12] uses gimbal angle and/or bearing information calculated from camera images. In this case, the integrated navigation method may not give an optimal navigation output since the inputs to the integration filter are processed outputs from raw measurements from the vision sensor. When the visual odometry is used for the integrated navigation system as in [13], the error of the navigation output from the vision navigation system increases with time. The integrated navigation method proposed in [14,15,16] uses the position and attitude, velocity or heading information from the vision navigation system. This integrated navigation method may not give a reliable navigation output when the number of landmarks in the camera image is not enough for the navigation output.

This paper proposes an inertial and vision integrated navigation method for poor vision environments, in which position and attitude outputs cannot be obtained from a vision navigation system due to the limited number of landmarks. The proposed method uses focal plane measurements of landmarks in the camera and INS outputs. Since there is no need to have navigation output from the vision navigation system, the proposed method can give integrated navigation output even when the number of landmarks in the camera is not enough for the navigation output. In addition to this, since the integration method uses raw measurements for integration filter, the navigation output may have better performance. In Section 2, a brief description of landmark-based vision navigation is given. The proposed integration method is presented in Section 3. Results of computer simulations and vehicle experiments are given in Section 4. The concluding remarks and further studies are mentioned in Section 5.

## 2. Landmark-Based Vision Navigation

**Figure 1:**
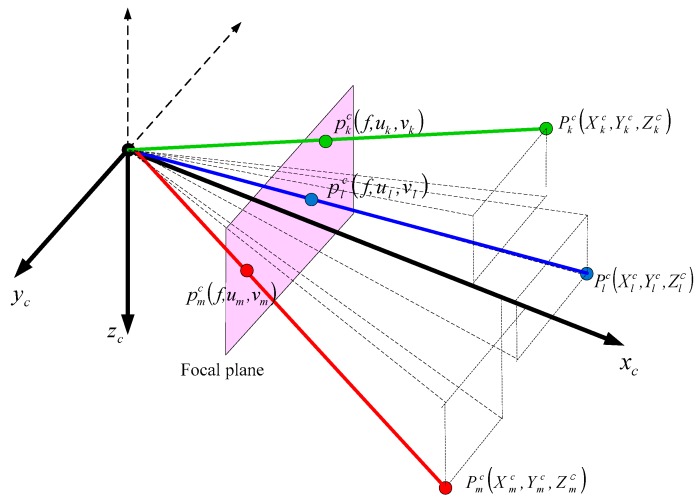
Landmark measurements in the vision navigation.

Vision navigation output is computed from the projected landmarks on the focal plane in landmark-based vision navigation [7,13,14]. Figure 1 shows projected landmarks on the focal plane when the pin hole camera model is adopted. The $x_{c}$ axis of the camera frame is aligned with the optical axis of the camera. The $y_{c}$ and $z_{c}$ axes are in the horizontal and vertical direction of the focal plane, respectively. The focal plane is placed at a distance of focal length, f, on the $x_{c}$ axis.

As shown in Figure 1, the landmark at position $P_{k}^{c}\left(X_{k}^{c},\;Y_{k}^{c},Z_{k}^{c}\right)$ is projected into the point $p_{k}^{c}\left(f,\;u_{k},v_{k}\right)$ on the focal plane in the camera frame. Equations (1) and (2) represent the relationship between the measurements on the focal plane and landmark coordinate values.

(1) $$u_{k}=f\frac{Y_{k}^{c}}{X_{k}^{c}}$$

(2) $$v_{k}=f\frac{Z_{k}^{c}}{X_{k}^{c}}$$

Equation (3) is the navigation equation to obtain navigation output from landmark measurement on the focal plane of the camera.

(3) $$P_{k}^{n}-P_{u}^{n}=r_{k}C_{b}^{n}C_{c}^{b}p_{k}^{c}$$

where the subscript k denotes index of landmarks and $P_{k}^{n}$ is a known position vector of the kth landmark. b, c and n denote the body frame, the camera frame and the navigation frame, respectively. $P_{u}^{n}$ is the vehicle’s three-dimensional position vector in the navigation frame. $r_{k}$ is the distance ratio of the projected landmark on the focal plane to the actual landmark in the camera frame. $C_{b}^{n}$ and $C_{c}^{b}$ are the direction cosine matrix from the body frame to the navigation frame and the direction cosine matrix from the camera frame to the body frame, respectively. Here, $C_{c}^{b}$ is a constant matrix since the camera is fixed to the body.

It can be seen from Equation (3) that at least three measurements are required in order to determine a navigation output of six variables, which are three-dimensional position and attitude [14]. In this paper, more than 0 and less than 3 landmarks are available in the poor vision environments.

## 3. Vision/INS Integrated Navigation System 

### 3.1. Vision/INS Integrated Navigation System

**Figure 2:**
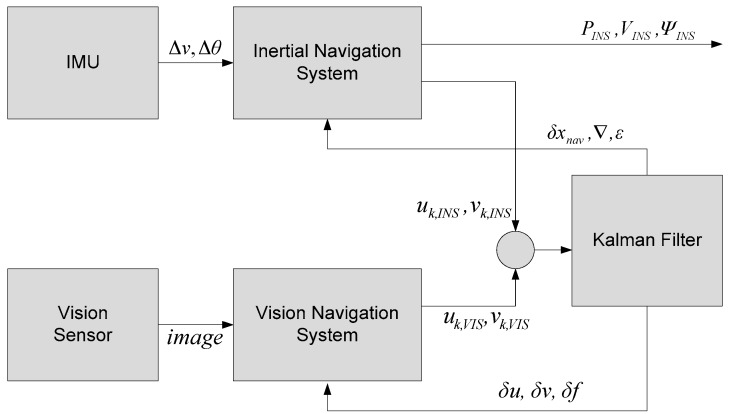
Proposed method of the vision/INS integrated navigation system.

Figure 2 describes the proposed method of a vision/INS integrated navigation system. The inertial navigation system computes vehicle’s position ($P_{INS}$), velocity ($V_{INS}$) and attitude (*Ψ_INS_*) from outputs of IMU (Inertial Measurement Unit) (Δ*_v_*, Δ*_θ_*). The vision navigation system gives projected landmark measurements on the focal plane ($u_{k,\;VIS}$, $v_{k,\;VIS}$). Kalman filter estimates the INS errors ($\delta x_{nav}$, ∇ and ε) and the vision sensor errors (δu, δv and δf).

### 3.2. Process Model of the Kalman Filter

INS error equation can be obtained by perturbing the navigation equation of the INS [17]. The process model based on the INS error equation and the sensor error equation for the proposed method is given in Equation (4).

(4) $$\dot{\delta x}\left(t\right)=F\left(t\right)\delta x\left(t\right)+w\left(t\right),\;\;w\left(t\right)\sim N\left(0,\;\;Q\left(t\right)\right)$$

where w(t) is process noise vector with covariance Q(t). Equation (4) can be rewritten into Equation (5).

(5) $$\left[\begin{array}{c} {\dot{\delta x}}_{nav}\\ {\dot{\delta x}}_{sen} \end{array}\right]=\left[\begin{array}{cc} F_{11} & F_{12}\\ 0_{9\times 9} & 0_{9\times 9} \end{array}\right]\left[\begin{array}{c} \delta x_{nav}\\ \delta x_{sen} \end{array}\right]+\left[\begin{array}{c} w_{nav}\\ w_{sen} \end{array}\right]$$

where $w_{nav}$ and $w_{sen}$ are the navigation parameter error vector noise and sensor error vector noise v, respectively. State vector δx is composed of navigation parameter error vector $\delta x_{nav}$ and sensor error vector $\delta x_{sen}$. $0_{m\times n}$ denotes an m by n zero matrix. The navigation parameter error vector is composed of position error, velocity error and attitude error of the INS as given in Equation (6).

(6) $$\delta x_{nav}={\left[\begin{array}{ccc} \delta P_{N} & \delta P_{E} & \delta P_{D} \end{array}\;\;\;\;\begin{array}{ccc} \delta V_{N} & \delta V_{E} & \delta V_{D} \end{array}\;\;\;\begin{array}{ccc} \varphi_{N} & \varphi_{E} & \varphi_{D} \end{array}\right]}^{T}$$

where δP, δV, and φ are position error, velocity error and attitude error expressed in the rotation vector, respectively. The subscripts N, E and D are the north, the east and the down axes in the navigation frame, respectively. The sensor error vector includes six inertial sensor errors and three vision sensor errors as in Equation (7).

(7) $$\delta x_{sen}={\left[\begin{array}{ccc} \nabla_{x} & \nabla_{y} & \nabla_{z} \end{array}\;\;\;\;\begin{array}{ccc} \varepsilon_{x} & \varepsilon_{y} & \varepsilon_{z} \end{array}\;\;\;\begin{array}{ccc} \delta u & \delta v & \delta f \end{array}\right]}^{T}$$

where ∇ and ϵ are accelerometer and the gyro error, respectively. δu and δv are errors of the coordinate values on the focal plane and δf is focal length error. The subscripts x, y and z denote roll, pitch and yaw direction in the body frame, respectively. Submatrix $F_{11}$ in Equation (5) is given in Equation (8).

(8) $$F_{11}=\left[\begin{array}{ccc} -\Omega_{en}^{n} & I_{3\times 3} & O_{3\times 3}\\ O_{3\times 3} & \Omega_{ie}^{n}+\Omega_{in}^{n} & f^{n}\times \\ O_{3\times 3} & O_{3\times 3} & -\Omega_{in}^{n} \end{array}\right]$$

where $\Omega_{en}^{n}$, $\Omega_{ie}^{n}$ and $\Omega_{in}^{n}$ are the skew-symmetric matrix of the vehicle’s craft-rate in the navigation frame, the skew-symmetric matrix of the earth rate in the navigation frame and the skew-symmetric matrix of the rotation rate of the navigation frame relative to the inertial frame represented in the navigation frame, respectively. $f^{n}\times$ is the skew-symmetric matrix of the vehicle’s specific force in the navigation frame. Submatrix $F_{12}$ in Equation (5) is given in Equation (9).

(9) $$F_{12}=\left[\begin{array}{ccc} O_{3\times 3} & O_{3\times 3} & O_{3\times 3}\\ C_{b}^{n} & O_{3\times 3} & O_{3\times 3}\\ O_{3\times 3} & -C_{b}^{n} & O_{3\times 3} \end{array}\right]$$

The accelerometer sensor error and the gyro error are modeled as random constants and are given in Equations (10) and (11), respectively.

(10) $$\dot{\nabla^{b}}=0$$

(11) $$\dot{\varepsilon^{b}}=0$$

The vision senor errors are also modeled as random constants and are given in Equations (12)–(14).

(12) $$\dot{\delta u}=0$$

(13) $$\dot{\delta v}=0$$

(14) $$\dot{\delta f}=0$$

### 3.3. Measurement Model of the Kalman Filter

The measurement equation for the Kalman filter is given in Equation (15).

(15) δz(t)=H(t)δx(t)+v(t),  v(t)~N(0, R(t))

where H(t) is the observation matrix and v(t) is the measurement noise vector with covariance R(t). The measurement vector δz(t) is given in Equation (16).

(16) $$\delta z={\left[\begin{array}{ccc} \delta u_{1}\; & \delta u_{2} & \cdots \end{array}\begin{array}{ccc} & \delta u_{n} & \delta v_{1} \end{array}\;\;\;\;\;\;\begin{array}{ccc} \delta v_{2} & \cdots & \delta v_{n} \end{array}\right]}^{T}$$

where $\delta u_{k}$ and $\delta v_{k}$ denote the differences between the INS-based estimates and measurements on the focal plane for the k-th landmark. n denotes the number of the landmarks on the focal plane of the camera. The INS-based estimates for each element in Equation (16) are calculated from position and attitude outputs of INS and the position information of the landmarks. The observation matrix is given in Equation (17).

(17) $$H\equiv \left[\begin{array}{ccc} H_{1} & H_{2} & H_{3} \end{array}\right]$$

Each sub-matrix in the observation matrix can be obtained from computing the Jacobian. The submatrix *H*_1_ is given in Equation (18).

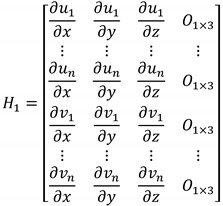
(18)
where ${\left[\begin{array}{ccc} x & y & z \end{array}\right]}^{T}$ is the position vector in the navigation frame. The submatrix $H_{2}$ is given in Equation (19).

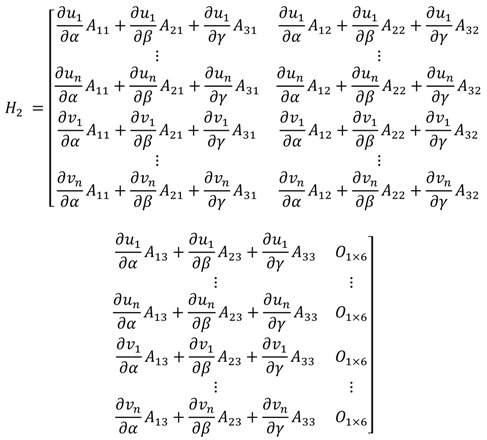
(19)
where ${\left[\begin{array}{ccc} \alpha & \beta & \gamma \end{array}\right]}^{T}$ is the attitude vector expressed in the Euler angle in the navigation frame. The attitude error in the process model Equation (6) is represented in the rotation vector, whereas the attitude error in Equation (19) is represented in the Euler angle. The relationship between the rotation vector and the Euler angle is expressed in Equation (26) [18]. The $A_{ij}\left(i=1,2,3,\;j=1,2,3\right)$ in Equation (19) are the same as those in Equation (20).

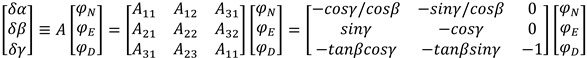
(20)

The submatrix $H_{3}$ is given in Equation (21).

(21) $$H_{3}=\left[\begin{array}{c} \begin{array}{ccc} -1 & 0 & -\frac{Y_{1}}{X_{1}}\\ \vdots & \vdots & \vdots \\ -1 & 0 & -\frac{Y_{n}}{X_{n}} \end{array}\\ \begin{array}{ccc} 0 & -1 & -\frac{Z_{1}}{X_{1}}\\ \vdots & \vdots & \vdots \\ 0 & -1 & -\frac{Z_{n}}{X_{n}} \end{array} \end{array}\right]$$

where ${\left[\begin{array}{ccc} X_{k} & Y_{k} & Z_{k} \end{array}\right]}^{T}$ is the position vector of the kth landmark in the camera frame and can be expressed in Equation (22).

(22) $$\left[\begin{array}{c} X_{k}\\ Y_{k}\\ Z_{k} \end{array}\right]=C_{n}^{c}\left[\begin{array}{c} x_{k}-x\\ y_{k}-y\\ z_{k}-z \end{array}\right]=\left[\begin{array}{ccc} C_{11} & C_{12} & C_{13}\\ C_{21} & C_{22} & C_{23}\\ C_{31} & C_{32} & C_{33} \end{array}\right]\left[\begin{array}{c} x_{k}-x\\ y_{k}-y\\ z_{k}-z \end{array}\right]$$

Then, $\left[\begin{array}{ccc} \partial u_{k}/\partial x & \partial u_{k}/\partial y & \partial u_{k}/\partial z \end{array}\right]$ in Equation (18) can be expressed in Equation (23).

(23) $$\left[\begin{array}{ccc} \frac{\partial u_{k}}{\partial x} & \frac{\partial u_{k}}{\partial y} & \frac{\partial u_{k}}{\partial z} \end{array}\right]=\left[\begin{array}{ccc} f\frac{Y_{k}}{X_{k}^{2}} & -f\frac{1}{X_{k}} & 0 \end{array}\right]\left[\begin{array}{ccc} C_{11} & C_{12} & C_{13}\\ C_{21} & C_{22} & C_{23}\\ C_{31} & C_{32} & C_{33} \end{array}\right]$$

And $\left[\begin{array}{ccc} \partial u_{k}/\partial \alpha & \partial u_{k}/\partial \beta & \partial u_{k}/\partial \gamma \end{array}\right]$ in Equation (19) can be expressed in Equation (24).

(24) $$\left[\begin{array}{ccc} \frac{\partial u_{k}}{\partial \alpha } & \frac{\partial u_{k}}{\partial \beta } & \frac{\partial u_{k}}{\partial \gamma } \end{array}\right]=\left[\begin{array}{ccc} -f\frac{Y_{k}}{X_{k}^{2}} & f\frac{1}{X_{k}} & 0 \end{array}\right]\left[\begin{array}{ccc} \frac{\partial X_{k}}{\partial \alpha } & \frac{\partial X_{k}}{\partial \beta } & \frac{\partial X_{k}}{\partial \gamma }\\ \frac{\partial Y_{k}}{\partial \alpha } & \frac{\partial Y_{k}}{\partial \beta } & \frac{\partial Y_{k}}{\partial \gamma }\\ \frac{\partial Z_{k}}{\partial \alpha } & \frac{\partial Z_{k}}{\partial \beta } & \frac{\partial Z_{k}}{\partial \gamma } \end{array}\right]$$

Also, $\left[\begin{array}{ccc} \partial v_{k}/\partial x & \partial v_{k}/\partial y & \partial v_{k}/\partial z \end{array}\right]$ in Equation (18) and $\left[\begin{array}{ccc} \partial v_{k}/\partial \alpha & \partial v_{k}/\partial \beta & \partial v_{k}/\partial \gamma \end{array}\right]$ in Equation (19) are expressed in Equations (25) and (26), respectively.

(25) $$\left[\begin{array}{ccc} \frac{\partial v_{k}}{\partial x} & \frac{\partial v_{k}}{\partial y} & \frac{\partial v_{k}}{\partial z} \end{array}\right]=\left[\begin{array}{ccc} f\frac{Z_{k}}{X_{k}^{2}} & 0 & -f\frac{1}{X_{k}} \end{array}\right]\left[\begin{array}{ccc} C_{11} & C_{12} & C_{13}\\ C_{21} & C_{22} & C_{23}\\ C_{31} & C_{32} & C_{33} \end{array}\right]$$

(26) $$\left[\begin{array}{ccc} \frac{\partial v_{k}}{\partial \alpha } & \frac{\partial v_{k}}{\partial \beta } & \frac{\partial v_{k}}{\partial \gamma } \end{array}\right]=\left[\begin{array}{ccc} -f\frac{Z_{k}}{X_{k}^{2}} & 0 & f\frac{1}{X_{k}} \end{array}\right]\left[\begin{array}{ccc} \frac{\partial X_{k}}{\partial \alpha } & \frac{\partial X_{k}}{\partial \beta } & \frac{\partial X_{k}}{\partial \gamma }\\ \frac{\partial Y_{k}}{\partial \alpha } & \frac{\partial Y_{k}}{\partial \beta } & \frac{\partial Y_{k}}{\partial \gamma }\\ \frac{\partial Z_{k}}{\partial \alpha } & \frac{\partial Z_{k}}{\partial \beta } & \frac{\partial Z_{k}}{\partial \gamma } \end{array}\right]$$

It can be seen from Equation (16) that the proposed method can provide an integrated navigation output even though the number of landmarks is not sufficient for a vision navigation output.

## 4. Computer Simulation and Experimental Result

The proposed method is verified through computer simulations and van tests.

### 4.1. Computer Simulation

**Figure 3:**
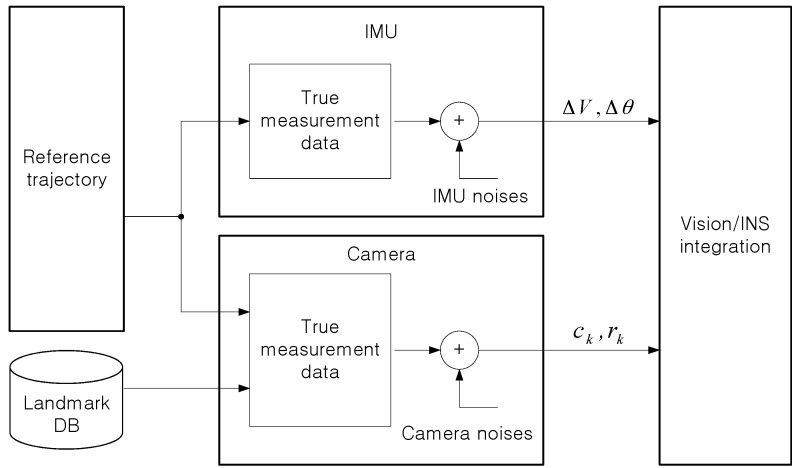
Scheme of simulation.

Computer simulations of the proposed integrated navigation method were carried out for a low medium-grade inertial sensor and a low-cost commercial camera. Figure 3 shows the scheme of the simulations. Reference trajectory and inertial sensor data were generated using MATLAB and INS tool box manufactured by GPSoft LLC. True camera measurement data of the landmarks were first generated using the pinhole camera model given in Equations (1) and (2). The camera measurement data on the focal plane of the landmarks were finally generated by adding noises into the true camera measurement data. Zero to ten landmarks to be observed on every image are placed by a random generator. The IMU measurement data were also generated by adding noises into the true IMU measurement data. Table 1 and Table 2 show the specifications of the IMU and the vision sensor for the simulation.

**Figure 4:**
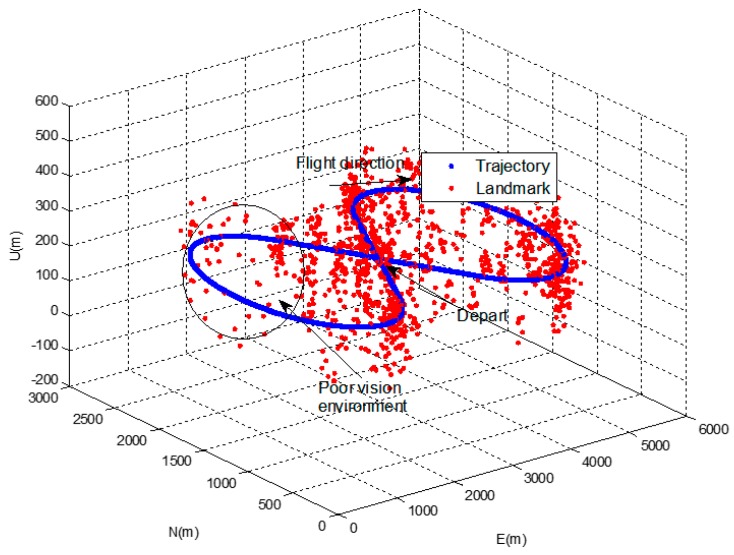
Vehicle trajectory and landmarks in simulation.

50 Monte-Carlo simulations were performed for an eight-shaped flight path with constant height as shown in Figure 4. Less than three landmarks were intentionally placed randomly in a specific area in order to create a poor vision navigation environment.

Results of the proposed method were compared with those of another integration method in [14]. In the integration method in [14], the outputs of the vision navigation system are position and attitude and state vector is given in Equation (27).

(27) $$\delta x={\left[\begin{array}{ccc} \delta P_{N} & \delta P_{E} & \delta P_{D} \end{array}\;\;\begin{array}{ccc} \delta V_{N} & \delta V_{E} & \delta V_{D} \end{array}\;\;\begin{array}{ccccccccc} \varphi_{N} & \varphi_{E} & \varphi_{D} & \nabla_{x} & \nabla_{y} & \nabla_{z} & \varepsilon_{x} & \varepsilon_{y} & \varepsilon_{z} \end{array}\right]}^{T}$$

The measurement vector is given in Equation (28).

(28) $$\delta z={\left[\begin{array}{cccccc} \delta P_{N} & \delta P_{E} & \delta P_{D} & \delta \alpha & \delta \beta & \delta \gamma \end{array}\right]}^{T}$$

As with the loosely coupled GPS/INS integrated navigation method, the method in [14] has redundancy in the navigation output. The vision navigation system can provide a stand-alone navigation output even when the INS and/or the integrated navigation system cannot provide a navigation output. However, as described in Section 2, the vision system cannot give navigation output when less than three landmarks are available on the focal plane. In this case, performance of integrated navigation system can deteriorate since the measurement update process cannot be performed in the integration Kalman filter. As shown in Equation (15), the measurement update process can be performed even when only one landmark is visible on the focal plane in the proposed method. Only the time update in Kalman filtering is performed when no landmarks are visible at all.

**Figure 5:**
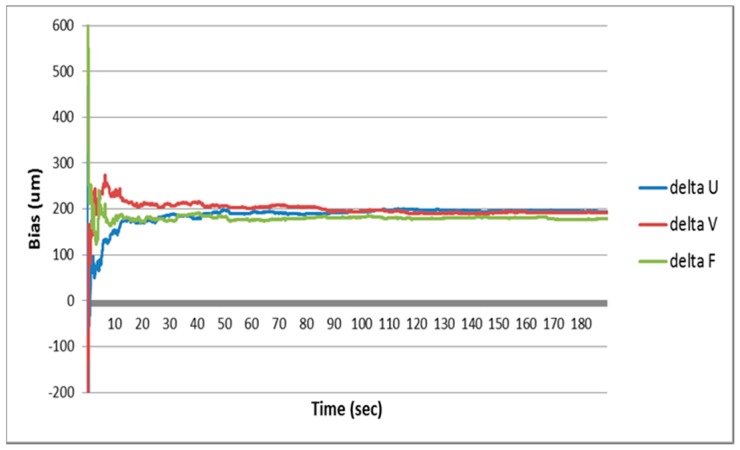
Camera sensor error estimation results of the simulation.

Figure 5 shows results of the estimated vision sensor errors of the proposed method in the simulation. It can be seen from the results that the vision sensor errors are well estimated and the performance of the vision navigation system is improved.

**Figure 6:**
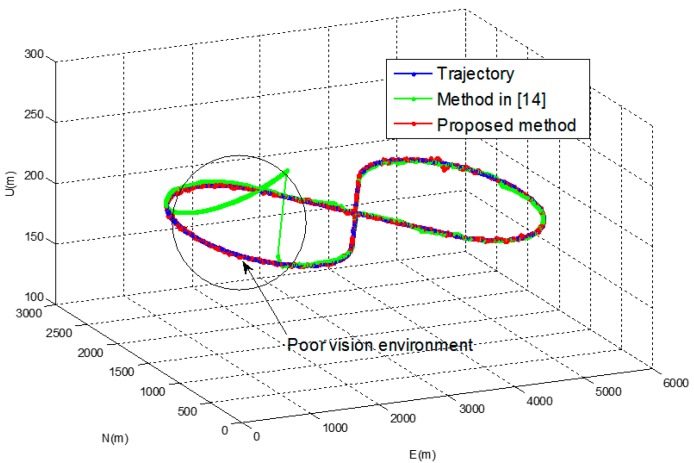
Navigation results of the simulation.

**Figure 7:**
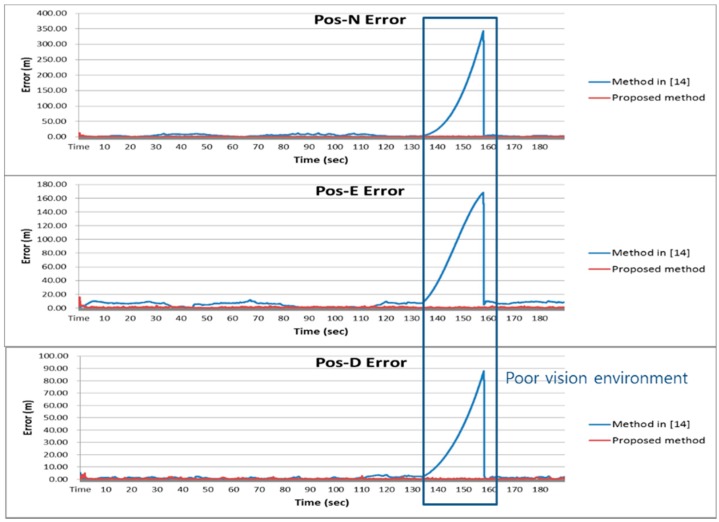
Position error of the simulation.

In Figure 6, navigation results of the proposed method are compared with those of the pure INS and the method in [14]. Figure 7 shows the position errors in the north, east and down direction of the proposed method and the method in [14].

Table 3 shows RMS errors for the pure INS, the method in [14] and the proposed method. It can be observed that error of the pure INS becomes large as the navigation operation continues. It can also be observed that the method in [14] gives relatively large navigation parameter errors in the area where the number of the landmarks are not enough for vision navigation output. The proposed method gives approximately 50 and 10 times better performance in the position and the attitude than the method in [14] in this area, respectively.

### 4.2. Van Test

**Figure 8:**
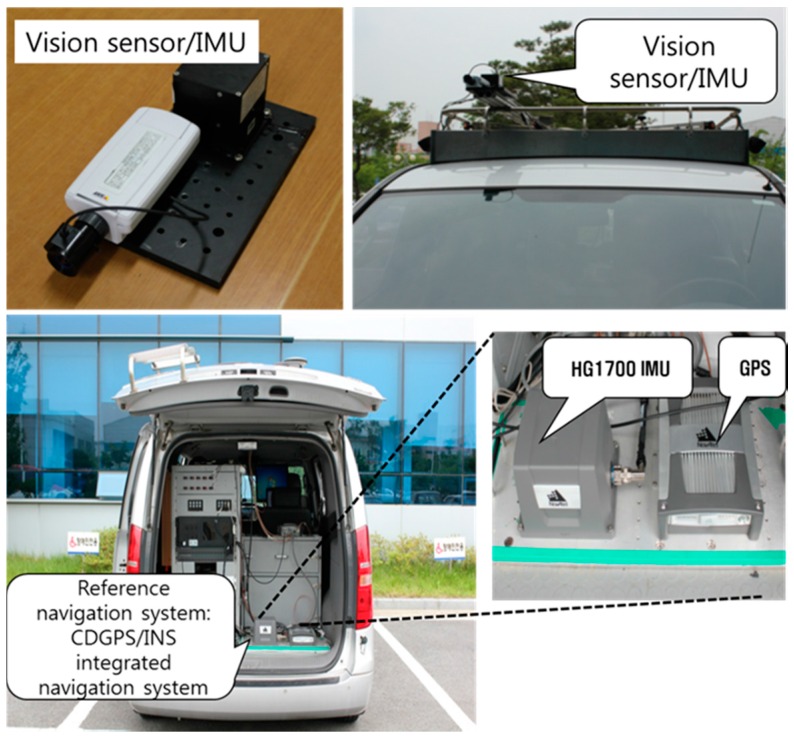
Experimental setup and reference navigation system.

Figure 8 shows the experimental setup and a reference navigation system. The experimental setup consists of a camera and an IMU and is installed on an optical bench. The reference navigation system, which is a carrier-phase differential GPS (CDGPS)/INS integrated navigation system, is installed together. Outputs of the reference navigation system are regarded as true values in the evaluation of the experimental results. A low-cost commercial camera and a micro electro mechanical system (MEMS) IMU given in Table 4 and Table 5 were used in the experiment. Database of the landmarks was made in advance with the help of large-scale maps and aerial photographs.

**Figure 9:**
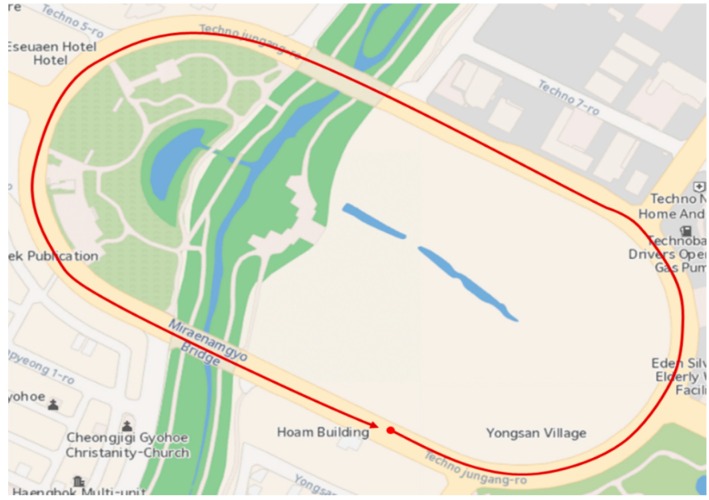
Vehicle’s reference trajectory in the experiment.

**Figure 10:**
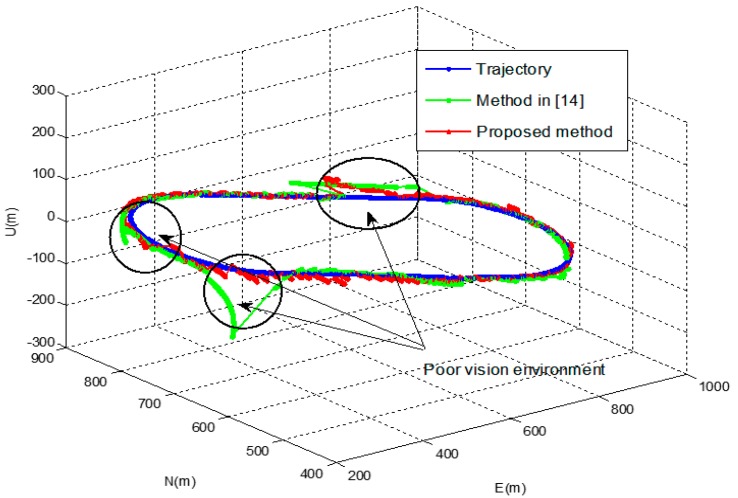
Navigation results of the experiment.

**Figure 11:**
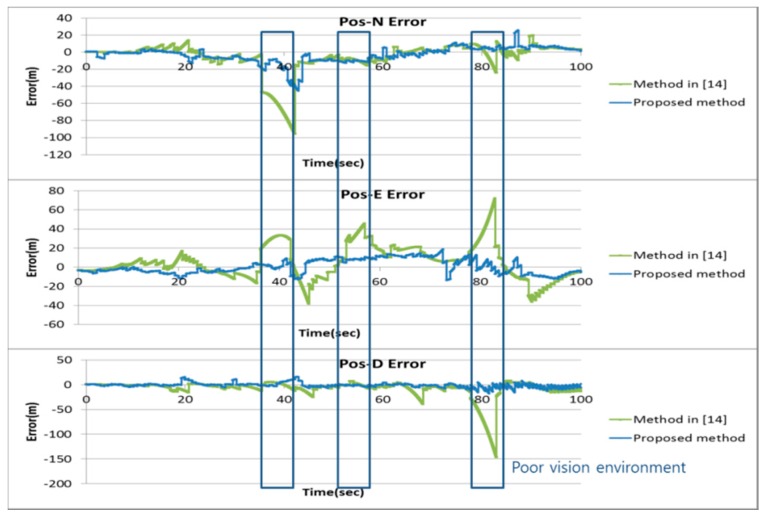
Position error of the experiment.

Figure 9 shows the position of the vehicle’s reference trajectory in the experiment. The results of the proposed method of the experiment were compared with those of the pure INS and the method in [14]. Figure 10 and Figure 11 show the navigation results and Table 6 shows the errors in the experiment. As with the results of the computer simulations, it can be seen from the experimental result that the proposed method provides reliable solutions with approximately 5 times better positioning performance than the method in [14] even in poor vision environments.

## 5. Concluding Remarks and Further Studies

This paper proposed an inertial and landmark-based vision integrated navigation method using focal plane measurements of landmarks. An integration model was derived to use the raw measurements on the focal plane in the integration Kalman filter. The proposed method has been verified through computer simulations and van tests. Performance of the proposed method has been compared with other integration method which used a vision navigation output, i.e., position and attitude output from a vision navigation system. It has been observed from the results that the proposed system gives reliable navigation outputs even when the number of landmarks is not sufficient for vision navigation.

An integration method to use continuous images to improve navigation performance and an integration model to efficiently detect and recognize landmarks will be studied. As future works, other filtering methods such as the particle filter and unscented Kalman filter, artificial neural network-based filtering and the application of a vision/INS integrated navigation system for sea navigation can be considered.

## Figures and Tables

**Table 1:** IMU specification and INS initial attitude error for simulation.

Specification	Description
Accelerometer bias	5 mg
Accelerometer random walk	0.1$\;m/s/\sqrt{h}$
Gyro bias	100°/h
Gyro random walk	0.5°$/\sqrt{h}$
Data rate	100 Hz
Roll Error	0.1°
Pitch Error	0.1°
Yaw Error	5.0°

**Table 2:** Vision sensor specification for simulation.

Specification	Description	Specification	Description
Focal length	25 mm	Avg. of focal lengh error	200 um
No. of horizontal pixels	4000	Avg. of horizontal optical axis coordinate error	200 um
No. of vertical pixels	3000	Avg. of vertical optical axis coordinate error	200 um
Field of view	90°	Focal lengh error (1σ)	200 um
Horizontal pixel pitch	8 um	Horizontal optical axis coordinate error (1σ)	200 um
Vertical pixel pitch	8 um	Vertical optical axis coordinate error (1σ)	200 um
Data rate	10 Hz		

**Table 3:** RMS navigation parameter error of the simulation.

Error		Pure INS	Method in [14]	Propsosed Method
Position error (m)	N	1026.88	18.59	0.99
	E	3350.72	15.90	1.12
	D	1370.55	5.34	0.45
Velocity error (m/s)	N	22.04	3.46	2.73
	E	22.09	2.88	3.07
	D	15.15	0.97	1.14
Attitude error (°)	Roll	0.69	2.02	0.06
	Pitch	0.65	2.44	0.37
	Yaw	8.42	3.14	0.63

**Table 4:** IMU specification and initial attitude error for the experiment.

Specification	Description
Manufacturer	Crossbow Ltd.
Accelerometer bias	10 mg
Accelerometer random walk	0.1 m/s/$\sqrt{h}$
Accelerometer scaling factor error	10,000 ppm
Gyro bias	3600°/h
Gyro random walk	1.0°/$\sqrt{h}$
Gyro scaling factor error	1000 ppm
Data rate	135 Hz
Roll error	0.61°
Pitch error	0.01°
Yaw error	4.30°

**Table 5:** Vision sensor specification for the experiment.

Specification		Description
Manufacturer		Axis Ltd.
Image sensor	Sensor type	CMOS-color
	No. of horizontal pixel	1280
	No. of vertical pixel	800
	Horizontal pixel pitch	3 um
	Vertical pixel pitch	3 um
Lens	Focal length	1.7 mm
	Field of view	99°
Data rate (frame rate)		Max 30 Hz (1.4 Hz is used in experiment)

**Table 6:** RMS navigation parameter error of the experiment.

Error		Pure INS	Method in [14]	Propsosed Method
Position error (m)	N	7110.95	9.29	6.50
	E	1228.32	16.44	6.47
	D	6973.98	15.34	3.25
Velocity error (m/s)	N	52.02	2.58	1.65
	E	166.06	4.13	1.87
	D	200.25	7.83	4.67
Attitude error (°)	Roll	32.54	4.04	2.31
	Pitch	20.82	3.53	2.91
	Yaw	53.05	5.61	4.68

## References

[1] Biezad D.J. (1999). Integrated Navigation and Guidance Systems.

[2] Groves P.D. (2008). Principles of GNSS, Inertial, and Multisensor Integrated Navigation Systems.

[3] Ehsan S., McDonald-Maier K.D. (2015). On-Board Vision Processing for Small UAVs: Time to Rethink Strategy. https://arxiv.org/abs/1504.07021.

[4] Groves P.D. (2013). The PNT boom, future trends in integrated navigation. Inside GNSS.

[5] Bryson M., Sukkarieh S. (2007). Building a robust implantation of bearing-only inertial SLAM for a UAV. J. Field Robot..

[6] Veth M.J. (2011). Navigation using images, a survey of techniques. J. Inst. Navig..

[7] Borenstein J., Everette H.R., Feng L. (1996). Where am I? Sensors and Methods for Mobile Robot Positioning.

[8] Thompson W.B., Henderson T.C., Colvin T.L., Dick L.B., Valiquette C.M. Vision-based localization. Proceedings of the 1993 Image Understanding Workshop.

[9] Betke M., Gurvits L. (1997). Mobile robot localization using landmarks. IEEE Trans. Robot. Autom..

[10] Chatterji G.B., Menon P.K., Sridhar B. (1997). GPS/machine vision navigation systems for aircraft. IEEE Trans. Aerosp. Electron. Syst..

[11] Scaramuzza D. (2011). Visual odometry-tutorial. IEEE Robot. Autom. Mag..

[12] George M., Sukkarieh S. Camera aided inertial navigation in poor GPS environments. Proceedings of the 2007 IEEE Aerospace Conference.

[13] Tardif J.P., George M., Laverne M. A new approach to vision-aided inertial navigation. Proceedings of the 2010 IEEE/RSJ International Conference on Intelligent Robots and Systems.

[14] Kim Y.S., Hwang D.-H. (2013). INS/vision navigation system considering error characteristics of landmark-based vision navigation. J. Inst. Control Robot. Syst..

[15] Yue D.X., Huang X.S., Tan H.L. INS/VNS fusion based on unscented particle filter. Proceedings of the 2007 International Conference on Wavelet Analysis and Pattern Recognition.

[16] Wang W., Wang D. Land vehicle navigation using odometry/INS/vision integrated system. Proceedings of the 2008 IEEE International Conference on Cybernetics Intelligent Systems.

[17] Meskin D.G., Itzhack Y.B. (1992). A unified approach to inertial navigation system error modeling. J. Guid. Control Dyn..

[18] Song G.W., Jeon C.B., Yu J. Relation of euler angle error and eotation vector error. Proceedings of the 1997 Conference on Control and Instrumentation.

